# Coxsackievirus A16-like particles elicit neutralizing antibody responses in mice

**Published:** 2011-01-10

**Authors:** Qingwei Liu, Kexia Yan, Yanfang Feng, Yicun Cai, Zhong Huang

**Affiliations:** 1 Key laboratory of Molecular Virology & Immunology, Institute Pasteur of Shanghai, Chinese Academy of Sciences, Shanghai 200025, PR China; 2 Biodesign Institute, Arizona State University, Tempe, Arizona 85287, USA

## 

Coxackievirus A16 (CVA16) is one of the major causative agents of hand, foot, and mouth disease (HFMD) currently prevalent in many countries and regions in the Far East. However, no vaccine for HFMD is yet available. Here we reported the production of CVA16 virus-like particle (VLP) and its immunogenicity in mice. Co-expression of P1 and 3CD of CVA16 in a baculovirus/insect cell system resulted in correct cleavage of P1 to yield subunit proteins VP0, VP1 and VP3. These three proteins were found to co-sediment by sucrose gradient analysis and assemble into VLPs. Mice immunized with VLPs generated high-titer CVA16-specific antibodies which efficiently neutralize live CVA16 *in vitro*. Collectively, our results indicate that CVA16-VLP can elicit potent neutralizing antibody responses and is therefore a promising vaccine candidate against CVA16 infection.

Coxackievirus A16 (CVA16) is one of the major causative agents of hand, foot, and mouth disease (HFMD) currently prevalent in many countries and regions in the Far East. However, no vaccine for HFMD is yet available. Here we reported the production of CVA16 virus-like particle (VLP) and its immunogenicity in mice. Co-expression of P1 and 3CD of CVA16 in a baculovirus/insect cell system resulted in correct cleavage of P1 to yield subunit proteins VP0, VP1 and VP3. These three proteins were found to co-sediment by sucrose gradient analysis and assemble into VLPs. Mice immunized with VLPs generated high-titer CVA16-specific antibodies which efficiently neutralize live CVA16 *in vitro*. Collectively, our results indicate that CVA16-VLP can elicit potent neutralizing antibody responses and is therefore a promising vaccine candidate against CVA16 infection.

